# Editorial: The Role of Obesity and Metabolic Syndrome in Couple Infertility

**DOI:** 10.3389/fendo.2021.784716

**Published:** 2021-11-10

**Authors:** Sara Marchiani, Lara Tamburrino, Nicole McPherson, Elisabetta Baldi

**Affiliations:** ^1^ Department of Clinical and Experimental Biomedical Sciences, University of Florence, Florence, Italy; ^2^ Department of Experimental and Clinical Medicine, University of Florence, Florence, Italy; ^3^ Faculty of Health and Medical Sciences, School of Biomedicine, University of Adelaide, Adelaide, SA, Australia; ^4^ Freemasons Centre for Male Health and Wellbeing, University of Adelaide, Adelaide, SA, Australia; ^5^ Robinson Research Institute, University of Adelaide, Adelaide, SA, Australia

**Keywords:** obesity, metabolic syndrome, infertility, reproduction, spermatozoa, oocyte

Metabolic syndrome (MetS) is a pathological condition characterized by abdominal obesity, insulin resistance, hypertension, and hyperlipidemia. It is estimated that in the USA MetS has reached an overall prevalence of more than 30% of adults older than 18 years, with incidences higher in some races and sociodemographic backgrounds (1). Together with obesity, the prevalence of MetS is increasing worldwide every year, with some of the biggest growths occurring in developing countries, gaining the terminology of a global pandemic (2).

MetS and obesity have a strong negative impact on reproductive function in both females and males causing hormonal imbalances and gonadal dysfunction. In women, both MetS and obesity are among the causes of polycystic ovarian syndrome (PCOS) (3) which, in turn, may result in alterations to endometrial receptivity. In men, obesity is related to hypogonadism and other sexual and reproductive dysfunctions, including alterations to gamete production and sperm function (4).

Several papers included in our research topic focused on PCOS, clarifying some aspects of the syndrome and giving some hints on possible treatments. Yang et al. demonstrated that serum prolactin levels are decreased in PCOS women and are positively associated with high-density lipoprotein cholesterol and, negatively, with body mass index (BMI), waist circumference, and reproductive hormone levels, likely contributing to the infertility problem of these women. Di Stasi et al. identified sex-hormone-binding globulin (SHBG) as a possible diagnostic parameter for the occurrence of non-alcoholic fatty liver disease associated with PCOS, as well as a possible marker of metabolic impairment in PCOS women. Barros de Melo et al. used an animal model to study the influence of high sucrose diet (HSD) administered during puberty on the ovarian hormonal milieu. They show that HSD determined MetS-like characteristics in these animals, which showed an increase in the prevalence of PCOS leading to ovarian dysfunction. Cao et al. showed that by evaluating three-dimensional (3D) genome interactions in ovary tissue from a PCOS mouse model, STON1 and FSHR genes were identified as potential targets for the PCOS-susceptible locus rs13405728, providing insights into the pathogenesis of PCOS. Two papers are related to therapeutic approaches for PCOS. Liu et al. reviewed published randomized control studies regarding insulin sensitivity and pregnancy rate in infertile PCOS women. Overall, these studies demonstrate that various non-surgical therapeutic strategies aimed to improve insulin sensitivity in PCOS women increase the possibility of successful pregnancy and that the improvement of insulin sensitivity might be even more important than reduction of BMI for ameliorating pregnancy rate. Zhu et al. employed a PCOS animal model to study the possible mechanisms of action of Chinese herbal medicine used in China to treat PCOS women. They found that the medicine decreases inflammation and improves insulin resistance in these animals by regulating intestinal flora.

Regarding obese/MetS men and reproductive function, Tančić-Gajić et al. evaluated testosterone levels on severely obese men affected by obstructive apnea. They found that both free and total testosterone levels were decreased in men with elevated apnea/hypopnea index, severe obesity, and MetS. Further, they found that obstructive sleep apnea is an independent determinant of serum testosterone concentrations in severely obese, highlighting the link between sleep quality and hormone production. Pini et al. reviewed the evidence of rodent dietary models of male obesity and reproductive dysfunction and the importance of assessed outcomes using a practical application approach such as Nutritional Geometry. Nutritional Geometry assesses outcomes of interest over an extended range of dietary macronutrient compositions rather than an individual macronutrient effect and, therefore, provides a promising tool for the development of evidence-based pre-conception nutritional guidelines for men. Finally, Zhao et al. assess the combined and independent impacts of both male and female body mass index (BMI) on cumulative pregnancy outcomes in a large Asian assisted reproduction cohort (15,972 couples). They found that both increasing female BMI and overweight males independently reduced cumulative pregnancy rates, with pregnancies rates further decreased if both partners were overweight, indicating a synergistic effect from couple overweight. They concluded that a couples approach to lifestyle change was warranted.

In summary, this Research Topic provides a large range of evidence that supports the collaborative efforts of endocrinologists, gynecologists, and reproductive biologists to continue to work together for the management of male and female reproductive health and the interplay of obesity and MetS.

## Author Contributions

All authors contributed to writing and approved the submitted version of this editorial by resuming the results of all scientific articles included in the Research Topic “*The Role of Obesity and Metabolic Syndrome in Couple Infertility*”.

## Conflict of Interest

The authors declare that the research was conducted in the absence of any commercial or financial relationships that could be construed as a potential conflict of interest.

## Publisher’s Note

All claims expressed in this article are solely those of the authors and do not necessarily represent those of their affiliated organizations, or those of the publisher, the editors and the reviewers. Any product that may be evaluated in this article, or claim that may be made by its manufacturer, is not guaranteed or endorsed by the publisher.
